# Acupuncture attenuates cognitive deficits and increases pyramidal neuron number in hippocampal CA1 area of vascular dementia rats

**DOI:** 10.1186/s12906-015-0656-x

**Published:** 2015-04-28

**Authors:** Fang Li, Chao-Qun Yan, Li-Ting Lin, Hui Li, Xiang-Hong Zeng, Yi Liu, Si-Qi Du, Wen Zhu, Cun-Zhi Liu

**Affiliations:** Acupuncture and Moxibustion Department, Beijing Hospital of Traditional Chinese Medicine Affiliated to Capital Medical University, 23 Meishuguanhou Street, Dongcheng District Beijing, 100010 China; Graduate School, Tianjin University of Traditional Chinese Medicine, No. 312, Anshan West Road, Nankai District, Tianjin 300193 China

**Keywords:** Vascular dementia, Cognitive function, Acupuncture, Hippocampus, Unbiased stereology

## Abstract

**Background:**

Decreased cognition is recognized as one of the most severe and consistent behavioral impairments in dementia. Experimental studies have reported that acupuncture may improve cognitive deficits, relieve vascular dementia (VD) symptoms, and increase cerebral perfusion and electrical activity.

**Methods:**

Multi-infarction dementia was modeled in rats with 3% microemboli saline suspension. Two weeks after acupuncture at *Zusanli (ST36)*, all rats were subjected to a hidden platform trial to test their 3-day spatial memory using the Morris water maze test. To estimate the numbers of pyramidal neuron, astrocytes, and synaptic boutons in hippocampal CA1 area, we adopted an unbiased stereology method to accurately sample and measure the size of cells.

**Results:**

We found that acupuncture at *ST36* significantly decreased the escape latency of VD rats. In addition, acupuncture significantly increased the pyramidal neuron number in hippocampal CA1 area (P < 0.05) and tended to decrease the number of astrocytes (P = 0.063). However, there was no significant change in the synaptic bouton number of hippocampal CA1 area in any of the groups (P > 0.05).

**Conclusions:**

These findings suggest that acupuncture may improve cognitive deficits and increase pyramidal neuron number of hippocampal CA1 area in VD rats.

## Background

Vascular dementia (VD) is a heterogeneous clinical disorder encompassing multiple vascular pathophysiological mechanisms occurring as a subtype of cerebrovascular disease (CVD) [1]. VD is the second most common cause of dementia after Alzheimer’s disease (AD) [2], and among the multiple types of VD, multi-infarction dementia (MID) is one of the most prevalent forms. Patients with VD generally experience a decline in cognitive function due to ischemic, ischemic-hypoxic, or hemorrhagic brain lesions caused by CVD and cardiovascular pathologic changes [3-5]. The hippocampus is considered one of the most important brain regions associated with learning and memory. In recent years, investigators have found that the hippocampus, especially hippocampal CA1 area, is particularly susceptible to ischemic insult [6,7].

Functional and morphological derangements in the hippocampus are among the most important factors contributing to cognitive dysfunction, such as the alterations of neurons, astrocytes and synapses. The neuron is the basic structural and functional unit of the nervous system. Neuronal death in the hippocampus is a major contributor to memory decline in the elderly [8-11]. In addition, the vulnerability of hippocampal CA1 pyramidal neurons plays a key role in the onset of cognitive impairment [12]. Astrocytes also perform critical functions in the brain such as promoting neovascularization, regulating neuronal activity, and supporting synaptogenesis and neurogenesis, which may influence recovery following ischemic injury. Changes in the astrocytes following ischemia may result from direct cellular injury or may occur in response to injury in other central nervous system (CNS) structures [13-15]. Synapses are the most sensitive and plastic structure in the CNS and are directly involved in the integration and transfer of information within the neural system. Synaptic plasticity, defined as activity-dependent changes in the strength of synaptic connections, is fundamental to the formation and maintenance of memory [16,17]. Currently, studies primarily examine the hippocampal volume, neurons, astrocytes, and synapses using qualitative and semi-quantitative methods [18,19]. However, these methods may not accurately quantify the number of changes in hippocampal neurons, astrocytes, and synapses. In 1988, Gundersen described a new stereological method to accurately and efficiently sample and measure the size of cells, a method that was employed in the examination of a wide range of cellular structures, including neurons, synapses, cancer cells, glomerular corpuscles, and ovarian follicles [20].

There is no effective medical or surgical treatment for VD at present. Acupuncture is a traditional Chinese medicine (TCM) method that has been used for both disease prevention and treatment for over 3000 years. Experimental studies have reported that acupuncture may improve cognitive deficits, relieve VD symptoms, and increase cerebral perfusion and electrical activity [21-24]. In the present study, we investigated the effect of acupuncture on memory performance and multiple cellular structures, including the neuron, astrocyte, and synaptic bouton numbers in hippocampal CA1 area, using the unbiased stereology method in VD rats.

## Methods

### Surgery and groups

All procedures were performed in accordance with requirements outlined by the Provisions and General Recommendations of Chinese Experimental Animal Administration Legislation and were approved by the China Academy of Chinese Medical Sciences Committee of Ethics on Animal Experiments. Seventy normal adult male Wistar rats (320–360 g) were used. All rats were group-housed (5 rats per cage) in plastic cages with wood-shaving bedding, a mean room temperature of 23 ± 2°C, 55 ± 5% humidity, and illumination from 7 AM to 7 PM daily. Rats were allowed free access to water and food. The animals were randomly divided into three groups: the normal group (n = 10), sham-operation group (n = 10), and surgery group (n = 40).

Ten milliliters of blood was drawn from the femoral artery of one male Wistar rat 36 h before surgery and stored at 37°C until a blood clot formed, then this rat was excluded from this test. The blood clot was then fragmented into 100–200-μm diameter sections, as measured by a micrometer. To induce focal ischemia in the rats, the surgery group was anesthetized with chloral hydrate (35 mg/100 g intraperitoneal). The neck was incised at ventral midline to expose the bifurcation of the right common carotid and external carotid arteries. A temporary clip was applied to the external carotid artery distal to the bifurcation, and 0.3 mL of a 3% microemboli saline suspension was injected into the internal carotid artery through disposable injection needles over 1–2 min. Rats in the sham-operation group were administered 0.3-mL normal saline in an identical manner. All rats were allowed 1 week to recover [25] and 7 animals were dead during the recovery period.

### Acupuncture manipulation

**Figure 1:**
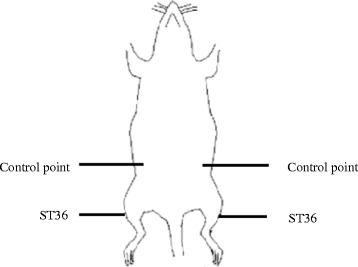
The detailed location of acupoints in this study.

One week after undergoing surgery, the surgery group was further randomly subdivided into three groups: an acupuncture group (n = 11); placebo-acupuncture group (n = 11); and an impaired group (n = 11). Animals in the acupuncture group were treated once daily over a 14-day period, with a rest day every 7 days, for a total 12 treatments. During acupuncture, the animals were awake and immobilized using special cages to minimize stress. For acupuncture, a small acupuncture needle, 0.3 × 40 mm (Hwato, China), was gently inserted in a depth of 5 mm in the *Zusanli* acupoint (*ST36*, 5 mm distal to the head of the fibula beneath the stifle and 2 mm lateral to the tibial tuberosity). The needles were twisted 2 times/s for 30 s. Animals in the placebo-acupuncture group received acupuncture at the hypochondrium (10 mm cranial to the iliac crest) bilaterally lasting for 30 s. The detailed locations of acupoints were shown as Figure 1. The remaining three groups (normal group, sham-operated group, and impaired group) were given the identical immobilization pattern and strength as rats in two treatment groups for the same 30 s duration.

### Behavioral analysis

After acupuncture treatment, all rats were subjected to a hidden platform trial to test their 3-day spatial memory using the Morris water maze (MWM) test as previously described by Morris [26]. A circular stainless steel tank (160-cm diameter, 50-cm height) was filled each morning with opaque water to a depth of 30 cm and 24 ± 1°C. The tank was artificially divided into four equal imaginary quadrants, northeast, northwest, southeast, and southwest, and a clear plexiglass platform (10-cm diameter, 28-cm height) was submerged 2 cm beneath the water surface in the center of the northeast quadrant. One day before the trial, each rat was trained and habituated to the water maze for 90 s without a platform. At the beginning of each trial, each rat was gently placed into the water at four different positions (never in the northeast quadrant) with its head facing the wall of the water maze, and was permitted 90 s to locate the hidden platform. The trial ended when the rat escaped onto the platform for 5 s, and the escape latency was recorded. If a rat failed to find the platform within 90 s, it was transferred onto the platform for 5 s by the investigator, and the escape latency was recorded as 90 s. At the end of the session, the rat was dried with a towel before being returned to its home cage [25-27]. Each trial was videotaped by a video camera suspended above the maze. The escape latency and swimming speed in each daily trial were automatically measured by an image analyzer (TopScan Lite Animal Behavior Analysis System; Clever Sys Inc., USA).

### Tissue preparation

All rats were anesthetized with 3.5% chloral hydrate (35 mg/100 g intraperitoneal) and perfused through the aorta with pre-cooled physiological saline, followed by 4% paraformaldehyde in phosphate buffer (PBS, 0.1 M, pH 7.4). The brains were immediately removed and post-fixed in 4% paraformaldehyde for 2–4 h, then dehydrated overnight in graded sucrose solutions (15%, 20%, and 30%, respectively) until completely submerged. The dehydrated brains were embedded in Tissue-Tek OCT (Optimal Cutting Temperature Compound; Sakura Finetek) under frozen conditions. Serial coronal sections measuring 50 μm were cut using a freezing microtome (Leica, Germany) and collected in sequence into 48-well tissue culture trays containing 500 μL of 4% paraformaldehyde. The tissue culture trays were sealed with parafilm to prevent evaporation of the fixative and stored at 4°C until further processing.

### Histochemistry and immunohistochemistry

The unbiased cell estimation was performed at every sixth section of hippocampal CA1 according to a systematic random sampling procedure. Approximately 114–132 consecutive sections were collected from the hippocampus in each animal and were subjected to staining for Nissl, glial fibrillary acidic protein (GFAP), or synaptophysin (SYN). For Nissl staining, sections were immersed in 0.01% toluidine blue (Hydratight, China) for 15–20 min at room temperature, dehydrated twice using a graded series of ethanol (70%, 80%, 90%, and 100%), made transparent using xylene, wet-mounted onto glass slides, and immediately sealed using neutral gum.

For GFAP and p38 immunohistochemistry staining, the sections were made transparent using 0.3% Triton X-100 (Sigma, USA) for 30 min at room temperature, followed by 3% H_2_O_2_ (ZSGB-BIO, China) at room temperature for 30 min to remove the endogenous peroxidase for 30 min, and were sealed for 1 h in 5% horse serum. Sections were transferred to a humid chamber for 1 h and incubated overnight at 4°C with polyclonal GFAP (Millipore, USA) or SYN antibody (Sigma, USA) diluted 1:400 or 1:200, respectively, in PBS containing 3% normal horse serum. The sections were then incubated for 1 h with the secondary antibody (goat anti-rabbit IgG; ZSGB-BIO, China) diluted 1:200 in PBS with 3% normal horse serum, followed by a 30 min incubation at room temperature in an avidin-biotinylated peroxidase solution (ZSGB-BIO, China) diluted 1:100 in PBS. Sections were examined for 5 min using 3,3′-diaminobenzidine (DAB, ZSGB-BIO, China), dehydrated twice in a graded series of ethanol, made transparent using xylene, wet-mounted onto glass slides, and immediately sealed with neutral gum.

### Unbiased stereology analysis

The sections were examined using a microscope (Leica DM4000B) equipped with a motor-driven stage to traverse the X- and Y-axes, and a microcator (Heidenhain, USA) to measure the Z-axis. A video camera (QI imaging, QICAM fast 1394) connected to a computer was attached to the microscope. Neurons and synaptic boutons were counted using a 100× oil immersion objective (NA, 1.3) and astrocytes using a 10× objective (NA, 0.25).

Four sections were randomly selected from each group to undergo examination using the unbiased stereology analysis system (MAC6000 system, Stereo Investigator 5.65, MBF, USA). Areal outline was confined to the pyramidal cell layer of hippocampal CA1 for further analysis based on a rat brain anatomic atlas (Paxinos and Watson, 1986). In order to accurately identify and count objects of interest in the microcator, the low power objective was replaced by a 4× objective (NA, 0.10). At each counting site, the mean thickness of the section (T) was carefully measured. The top (upper surface) of the tissue section was defined as the first cell coming into focus and the bottom (lower surface) of the tissue section as the last cell coming into focus. The distance between the top and bottom was defined as T. In the present study, the T was 21 μm. The numbers of pyramidal neurons, astrocytes, and synapses in hippocampal CA1 were estimated using the optical fractionator method. The optical fractionator method is based on a properly designed systematic random sampling method that by definition yields unbiased estimates of the population number [28,29]. The number of cells was determined by measuring three sampling fractions: the section sampling fraction (ssf); area sampling fraction (asf); and thickness sampling fraction (tsf). The estimated total number of cells (N) in hippocampal CA1 was estimated by multiplying the reciprocals of the sampling fractions to total number of counted cells (∑_Q_^−^) according to the following equation [30]:

$$ \mathrm{N}=1/\mathrm{Volume}\ \mathrm{Fraction}\times {\sum_Q}^{-}=1/\mathrm{s}\mathrm{s}\mathrm{f}\times 1/\mathrm{a}\mathrm{s}\mathrm{f}\times 1/\mathrm{t}\mathrm{s}\mathrm{f}\times {\sum_Q}^{-} $$

Sampling was optimized to produce a coefficient of error (CE) less than the observed biological variability, and the CE values remained less than or equal to 0.2, which was automatically calculated by the Stereo Investigator 5.65 software and deemed appropriate for the present study. A summary of the experimental stereological parameters and optical fractionator counting results are shown in Table 1.

**Table 1:** **Experimental unbiased stereological parameters**

**Parameters**	**Pyramidal neuron**	**Astrocyte**	**Synaptic bouton**
Counting frame size (μm^2^)	19.6 × 19.6	100 × 100	4 × 4
Sampling grid size (μm^2^)	80 × 80	200 × 200	120 × 160
Disector height (H, μm)	12	12	9
Mean final section thickness (T, μm)	21	21	21
ssf	6	6	6
asf	0.06	0.25	0.0008
tsf	0.57	0.57	0.43

ssf, section sampling fraction; asf, area sampling fraction; tsf, thickness sampling fraction.

### Statistical analysis

All statistical analyses were performed by an observer blinded to the experimental group. All data were analyzed by one-way ANOVA. When appropriate, post-hoc comparisons were assessed using the LSD test (equal variances assumed) or Dunnett’s T3 test (equal variances not assumed). Statistical analysis was performed using SPSS (version 16.0, SPSS Inc., Chicago, Illinois, USA). *P* values less than 0.05 were considered statistically significant.

## Results

### Behavioral testing

**Figure 2:**
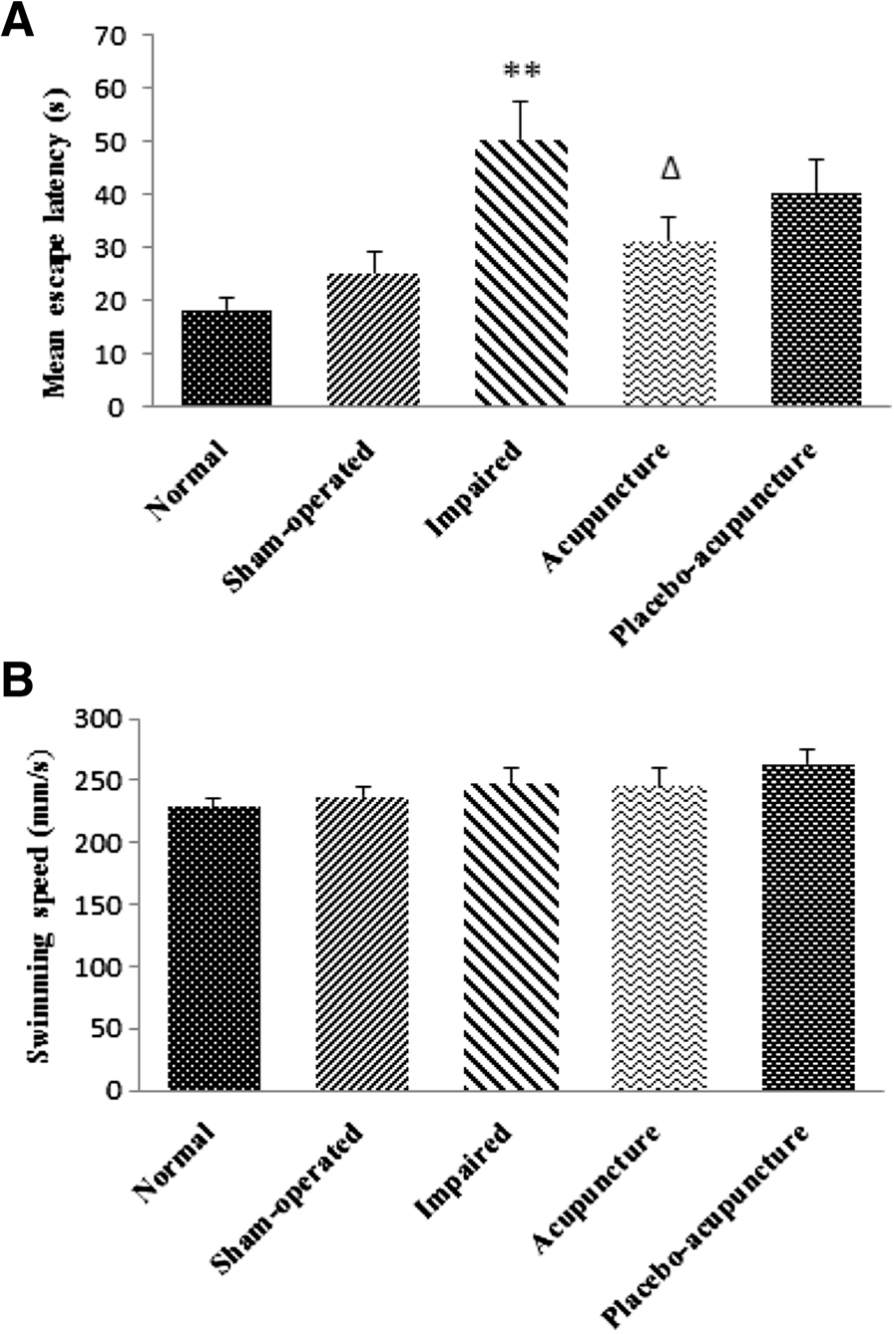
Performance in the MWM test of each group over 3 days. **(A)** Total escape latency of each group over 3 days. **(B)** Total swimming speed of each group over 3 days.

To investigate the effects of acupuncture on spatial learning in VD rats, the MWM test was performed, and the learning ability of the animals was determined by measuring the escape latency (Figure 2A). Compared to the normal group, the total escape latency was significantly prolonged in the impaired group (P < 0.01, Figure 2A). After treatment, the acupuncture group showed a significant decrease in the total escape latency compared to that in the impaired group (P < 0.05, Figure 2A). However, there were no significant differences between the placebo-acupuncture and impaired groups (Figure 2A). The total swimming speed showed no statistical differences among the five groups (P > 0.05, Figure 2B).

### Morphologic changes

**Figure 3:**
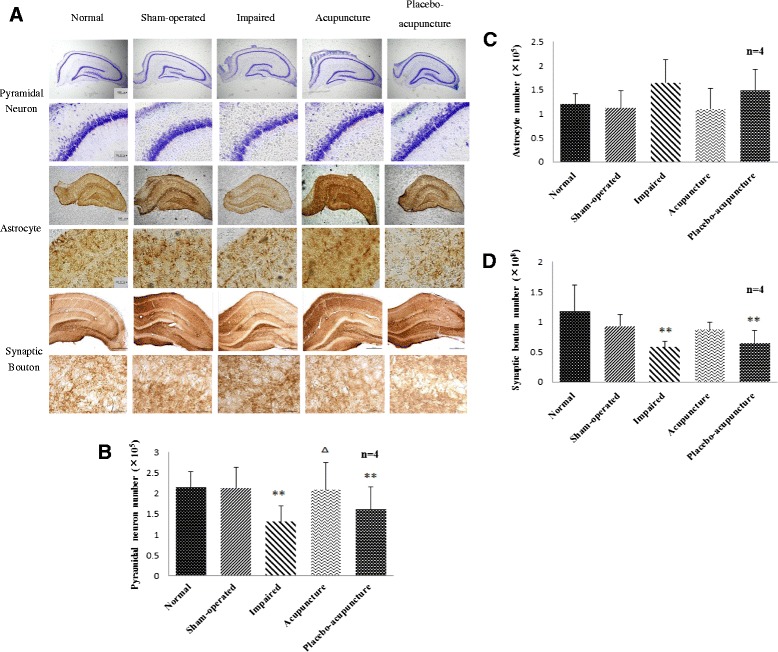
Unbiased stereological results for the number of pyramidal neurons, astrocytes, and synaptic boutons in hippocampal CA1 area. **(A)** Representative sections of Nissl staining for pyramidal neurons, GFAP immunostaining for astrocytes, and SYN immunostaining for SYN-positive synaptic boutons (For pyramidal neurons and astrocytes, above: 4 × magnification, scale bar = 200 μm; Below: 40 × magnification, scale bar =20 μm. For SYN-positive synaptic boutons, above: 5 × magnification, scale bar = 500 μm; Below: 100 × magnification, scale bar =20 μm.). **(B–D)** Changes in the number of pyramidal neurons, astrocytes, and synaptic boutons in each group.

In present study, we estimated the numbers of pyramidal neurons, astrocytes and synaptic boutons, in hippocampal CA1 using the optical fractionator method of unbiased stereological analysis. The pyramidal neuron number in hippocampal CA1 was significantly decreased in the impaired group compared to that in the normal group (P < 0.01, Figure 3B). After treatment, the acupuncture group showed a significant increase in the pyramidal neuron number compared to the impaired group (P < 0.05, Figure 3B). However, there was no significant difference in the pyramidal neuron number between the placebo-acupuncture and impaired groups (Figure 3B). These results indicate that acupuncture may increase the number of pyramidal neurons in hippocampal CA1 of the VD rat.

There were no significant differences in the astrocyte number in hippocampal CA1 among the five groups (P > 0.05, Figure 3C). Although there were no significant differences between the acupuncture and impaired groups in the astrocyte number, a decreasing trend was observed in the acupuncture group (P = 0.063, Figure 3C). This demonstrates that acupuncture may influence the astrocyte number in hippocampal CA1 in VD rats to some extent.

The number of synaptic bouton (SYN-positive bouton) in hippocampal CA1 was significantly decreased in the impaired group compared to the normal group (P < 0.01, Figure 3D). There were no significant differences between the acupuncture and impaired groups in for the synaptic bouton number, although a slight increase was observed in the acupuncture group (P = 0.116, Figure 3D). Representative sections showing the Nissl, GFAP, and SYN staining are shown in Figure 3A.

## Discussion

Previous studies reported the neuroprotective effect of acupuncture for VD rats in improving cognitive function [31,32], increasing glucose metabolism [23], reducing oxidative stress damage [22,25,33], and exerting anti-apoptotic effect [24,34]. Furthermore, our previous clinical trials suggested that acupuncture may be useful in relieving symptoms of VD to some extent [34,35]. Based on these clinical trials, we choose the representative acupoint *ST36* to explore the biological mechanisms of acupuncture for improving cognitive function in VD rats.

Decreased cognition is recognized as one of the most severe and consistent behavioral impairments in dementia. MWM is a standard method to assess spatial leaning ability in rodents. Recent studies have reported that VD animal exhibit significant learning and memory deficits during the MWM test, with a longer escape latency reported [36,37]. In this experiment, we found that acupuncture at *ST36* significantly decreased the total escape latency of VD animals, but placebo-acupuncture had no effect on the total escape latency. Although there was no statistical difference between the acupuncture and placebo-acupuncture groups, the effect of placebo-acupuncture was inferior to that of acupuncture. Our findings indicate that acupuncture caused a greater improvement in the learning and memory ability compared to placebo-acupuncture in VD rats, which is consistent with previous studies [22].

The relationships between neuronal loss in the hippocampus and cognitive impairment warrant exploration [38]. The Nissl body is a structure unique to neurons, and the density of Nissl staining in the neuronal cytoplasm is used to evaluate neuronal damage [39-41]. Cognitive deficits have been associated with damage in hippocampal CA1 [6,20], and permanent occlusion of the bilateral common carotid arteries in animals induces significant pyramidal neuron loss in hippocampal CA1 area [42]. In addition, chronic cerebral hypoperfusion triggers reactive astrocytosis with detectable morphological signs and accumulated GFAP in active astrocytes [43]. GFAP, one of the most highly synthesized proteins in brain, is widely used to study the active state of astrocytes and may play a role in the ischemic process [13]. GFAP-knockout mice show greater susceptibility to ischemic injury [44]. SYN is a presynaptic-specific marker that is highly concentrated at presynaptic boutons and closely associated with synaptic plasticity. Synaptic plasticity encompasses a great variety of changes in synaptic function and structure. Functional synaptic plasticity includes two major forms, long-term potentiation (LTP) and long-term depression (LTD), while structural synaptic plasticity mainly represents synaptogensis and morphologic change in synapses [45]. SYN-positive boutons are also a sensitive indicator of cognitive deficits [35]. Synapse loss has been closely correlated with cognitive impairment in dementia [46]. Notably, Calboum [47] found that stereological measurement of total neuron number in hippocampal CA1 did not show any significant age-related change. After transient middle cerebral artery occlusion (tMCAO), there was no evidence of neuron loss or change in the general synaptic transmission and presynaptic plasticity in hippocampal CA1 [48].

In the current study, we found that acupuncture treatment significantly increased pyramidal neuron number in hippocampal CA1 area and improved the cognitive performance of VD rats. These results are similar to those of previous studies showing that repetitive transcranial magnetic stimulation (rTMS) improved both the morphology and the learning and memory ability of VD rats [43,49]. In addition, our results preliminarily confirmed that the effect of acupuncture on improving cognitive dysfunction is likely related to an increased pyramidal neuron number in hippocampal CA1 area of VD rats. Although the effect of increased GFAP immunoreactivity during hypoperfusion is not fully understood, it is well known that astrocyte changes are the most dramatic response of the brain to ischemic injury and may have a critical impact on the evolution and outcome of the ischemic lesion [13]. Vicente et al. found that 10 weeks of chronic hypoperfusion caused a significant GFAP increase in the hippocampus, confirming that astrocytes were activated during chronic ischemia [50]. In the present study, we found that acupuncture tended to decrease the number of astrocytes in hippocampal CA1 area; that is, acupuncture inhibited astrocyte activation and proliferation to some extent. However, the mechanisms underlying of the impact of acupuncture on astrocytes, especially the signaling pathway of astrocyte activation, requires further investigation. Synapse loss was closely correlated with cognitive impairment, but we observed that acupuncture had no significant effect on the synaptic bouton number. Our earlier study showed that acupuncture could significantly restore the impaired LTP and improve cognitive deficit in MID rats [32]. It has been reported that electroacupuncture (EA) could reduce behavior deficit and long-term potentiation (LTP) in VD model rats [51]. Similarly, a previous study showed that EA could improve learning and memory performance by enhancing LTP in diabetic rats with cerebral ischemia [52]. Potentially, the regulation of hippocampal CA1 synapses by acupuncture may be related to functional synaptic plasticity (LTP or LTD), rather than structural synaptic plasticity. Above studies suggested that acupuncture could improve behavioral performance after cerebral insults and enhance the hippocampal LTP. However, due to insufficience in evidence, its underlying mechanisms remain unclear. Further research into the hippocampal LTP involved in acupuncture-induced improvement of cognitive deficits is warranted.

There were no statistical differences between the acupuncture and placebo-acupuncture groups in the behavioral and morphologic analyses. However, our previous study indicated that the effect of acupuncture was superior to that of placebo-acupuncture on behavioral tests [25]. This conflict may reflect differences in the administration of acupuncture and the needling duration in both studies. Our earlier study performed acupuncture at *Tanzhong (CV17), Zhongwan (CV12), Qihai (CV6), ST36* and *Xuehai (SP10)* [25]; in contrast, in the present study, treatment was performed at *ST36* alone. Furthermore, acupuncture was performed for 14-day duration, which is shorter than the treatment duration in the earlier study. Therefore, the efficacy of acupuncture at a single acupoint over 14 days may be inferior to treatment performed at multiple acupoints over 21 days. Further studies using the more effective multiple acupoint protocol are needed to enhance our understanding of the effects of acupuncture. Moreover, a more suitable placebo-acupuncture should be adopted to reflect the exact effects of acupuncture. The present study only focused on the synaptic number in hippocampal CA1. Thus, further studies are warranted exploring functional synaptic plasticity (LTP or LTD).

## Conclusion

Acupuncture at *ST36* increase pyramidal neuron number in hippocampal CA1 area which may promote the recovery of cognitive function in VD rats. However, we didn’t identify the increase of different neuronal types in present study, although we observed the increase of pyramidal neuron number. Moreover, whether or not the increase in cell proliferation is related to the increase of pyramidal neuron number in hippocampal CA1 area for acupuncture? In the future study, we will explore the more detail molecular mechanism of the acupuncture neuroprotective effect.
